# Supplementary material for: Nitrogen and phosphorus fertilization consistently favor pathogenic over mutualistic fungi in grassland soils
Source: Nat Commun. 2021 Jun 9;12:3484. doi: 10.1038/s41467-021-23605-y (PMC8190096; doi:10.1038/s41467-021-23605-y)
Supplement: Supplementary file 1 — Supplementary Information [file 41467_2021_23605_MOESM1_ESM.pdf]

SUPPLEMENTARY INFORMATION

Supplementary Methods

<u>Rationale for local and global drivers of fungal guilds</u>

Arbuscular mycorrhizal fungi (AMF): In general, we expect AMF to increase in abundance in situations where plants are not carbon (C) limited and when they are likely to benefit from AM-services[1], including where diffusion-limited nutrients [in particular phosphorus (P)] are scarce, where pathogens are abundant, and when plants experience drought[2]. High natural availability of P, or P addition is likely to support lower abundances of AMF[3] as less C is allocated to AMF. Nitrogen (N) addition and a high soil N:P, on the other hand, may promote AMF as it likely results in greater P-limitations[4], but when N is added together with P, or when N-addition increase the abundance of nitrophilic plants, such as C_3 grasses that are likely poorer hosts to AMF, AMF abundance will likely decline[4–6]. Soils with higher C:N may also support a lower abundance of AMF as N and not P likely limits plant growth, whereas AMF abundance is expected to higher in highly weathered mineral soils[7,8]. We also expect AMF abundance to be greater in grasslands that have higher photosynthetic active radiation (PAR) due to greater C-assimilation[8] and to increase with temperature[7,9] and potential evapotranspiration[7,10]. Because AMF may help with drought tolerance[2,11] and due to the relationship between precipitation and potential evapotranspiration (PET), we expect the relationship between AMF abundance and precipitation to be negative so long as it does not reduce the ability of plants to allocate C to AMF. Likewise, AMF abundance is expected to correlate positively with pH[10,12,13], possibly due to metal toxicities at lower pH. AMF abundance is assumed to respond positively to increased plant biomass given that larger plants can allocate more C belowground than smaller plants, although this will depend on the specific C-allocation to AMF, which may not necessarily correlate with aboveground biomass but be more dependent on the specific plant community[14]. Due to potential differences in host quality, AMF may benefit more by forbs than grasses[15,16], although there are many exceptions to this rule including many C_4-grasses that tend to be good hosts[15]. Therefore, we expect most effects of climate and soil conditions on both soil and root AMF abundance to be mediated by plant responses.

Plant pathogens: Like AMF, interactions between plants and fungal pathogens are complicated and depend on both host and pathogen responses to environmental conditions[17]. Also, most work to date regarding pathogens has been conducted on cultivated plants and less is known about pathogens in natural communities, although recent work has highlighted their potential role in maintaining plant diversity[18–20]. One pattern that seem pretty clear across studies is that pathogens often thrive in resource-rich environments[21–23]. This effect is likely greater with N than P, because P can sometimes enhance plant vigor, which would decrease the susceptibility to pathogens[24,25]. As such, we expect pathogen abundance to be promoted by N but be reduced by P, and for pathogens to be more problematic when soil N:P is high and soil C:N low. Responses to other environmental conditions are more difficult to predict as responses seem to depend on the particular pathogen involved and also the degree of host stress. For example, higher precipitation can enhance seedling mortality from fungal pathogens[26] by pathogens that thrive in wetter soils[27], but drought-stress can make plants more susceptible to disease, and a subset of fungal pathogens are most problematic at low soil moisture[27]. This suggest that the effect of precipitation on pathogen abundance may depend on the degree of stress and what fungal pathogens are present. Finally, if pathogens are species-specific[18] and if pathogen accumulation is density dependent[19,28,29], then we expect negative relationships between pathogen abundance and plant diversity.

Saprotrophs: In general, saprotrophs are going to respond to shifts in litter productivity (substrate) and tissue stoichiometry so we expect responses to be indirectly moderated by plant responses. Much research has been devoted to the effect of N-addition on decomposition due to implications for C-sequestration. It seems like N-responses are varied and can change over time, depend on the saprotrophic community, litter quality, soil fertility and amount and source of N added[30–34], making predictions challenging. In addition, very little is known about P and saprotrophs. If lower quality soil organic carbon promotes decomposers[35], saprotroph abundance should be suppressed by nutrient additions (even though decomposition rate may go up[36]), and correlate negatively with soil nutrient availability but positively with soil C:N. We also expect plant biomass to be higher in warmer, wetter climates, and this greater detritus

production ought to stimulate saprotrophs[14]. Based on previous work, we also predict saprotroph abundance to be lower at higher soil pH[37,38]. Finally, if graminoids decompose slower than forbs[36] and if saprotroph abundance is promoted by a lower litter quality, then we expect that saprotrophs would be promoted by grasses and suppressed by forbs.

Data sources and methods

We used soil fungal community data, soil chemistry and plant community characteristics collected from 25 temperate-zone grassland sites in the multiple-nutrient addition experiment established as part of the Nutrient Network (NutNet) experiment. This experiment uses standardized treatments that are applied yearly in 5 x 5 m plots, including N, P and N+P, each added at 10 g m^{-2} yr^{-1}. For the purpose of this study, we restricted our analyses to these treatments as that is what we had fungal sequencing data for. At each site, treatment plots are nested within blocks in a fully randomized design, with each site replicating the experiment in at least 3 blocks.

For detailed descriptions of methods associated with NutNet experiments see Borer et al.[39,40]. Briefly, soil properties (pH, C, N, P, Ca, Mg, S, Na, Zn, Mn, Fe, Cu, B) were measured at the beginning of the experiment, before treatments were applied and soil and root samples were collected for DNA extraction and microscopy. In every plot, two 2.5 cm diameter by 10 cm depth soil cores were collected at the peak of the growing season. Samples for chemical analysis were homogenized through a 2mm sieve and air dried. Dry combustion GC analysis (COSTECH ESC 4010 Element Analyzer, University of Nebraska, Lincoln, NE, USA) was used to assay for %N and %C, and all other elements were assessed in an external lab (A&L Analytical Laboratory, Memphis, TN, USA).

Aboveground biomass per plant functional group and species cover was recorded yearly at peak biomass in each plot during the experiment. The aboveground biomass of all plants rooted within two 0.1 m^2 (10 x 100 cm) strips in each experimental plot was clipped, separated into functional groups, dried at 60°C for 48 hrs, and weighed to the nearest 0.01 g. Plant richness was quantified by visually estimating the percent cover of each species to the nearest 1% in a permanently marked, 1 x 1 m subplot within each 25 m^2 plot.

Soil cores for the fungal analysis were collected after 1-4 years of fertilization (see Leff et al[41] for detailed description of soil sampling, DNA extraction, amplification and bioinformatics). Root biomass[42]and pH were also measured in these cores, and we scored roots present for overall colonization by AMF using the gridline intersect method[43] on trypan blue stained roots.

Plant and soil data used in these analyses were retrieved from the NutNet database on June 3, 2020, but where restricted to years of sampling except for the mediation test where we used average values from control plots spanning 0-4 years since establishment wherever possible. We downloaded climate descriptors from Worldclim v2[44] to represent long-term historical climate differences among sites, and focused on six non-correlated variables: year average temperature, mean diurnal range, temperature seasonality (standard deviation x 100), total annual precipitation, precipitation seasonality (coefficient of variation), precipitation of warmest quarter and precipitation of coldest quarter. Climatic descriptors were only available at the site level. We also recorded latitude, altitude and elevation of each site to accommodate the spatial correlation used by Worldclim to interpolate the climatic parameters.

Climatic and location predictors were not used for the local models, and edaphic and plant community descriptors were aggregated at the site level for the global models. For the global analysis, we restricted the analysis to control plots to capture the expected global trends in unmanipulated systems and used long-term averages of vegetation predictors to minimize effects of year-to-year variation. In particular, we represented biomass and litter at a site level as across-year averages of the control plots biomass and litter, respectively, soil properties were the mean value of the control plots at the beginning of the experiment, and guild abundances were the mean value of the control plots when the samples were collected.

We estimated biomass and biodiversity measurements of the whole plant community and of each functional group. For the global analysis, we accumulated all the species observed across 5 years in the control plots in each site. Using this aggregated species list we estimated the site-level richness, Faith's phylogenetic diversity (PD) [45], mean phylogenetic distance (MPD) and mean nearest phylogenetic distance (MNTD). We repeated the biomass and diversity measures using species that belong to different functional groups (grasses, legumes and forbs)

and obtained biomass and biodiversity estimates by functional group. For the local models, we used biomass and cover data of the year when the fungi soil samples were collected, and estimated the same predictors: richness, PD, MPD, and MNTD, each using the whole community and each functional group. MPD and MNTD were not estimated for legumes, as legumes were absent in some sites and plots. Similar to Leff et al.[41], we estimated the Jaccard dissimilarity between each plot and the corresponding control plot of the given block in each site. We included litter mass as a predictor for saprotrophs.

Selecting fungal guild predictors

Local models aimed to test the effect of nutrient addition on fungal guilds, while global models aimed to find drivers of guild abundances at a global scale. In both cases, we used similar approaches. For each level of analysis and each guild we fitted the most parsimonious model using each set of predictors: pre-treatment soil conditions, pH and root biomass collected at the same time as the fungal guild (local only), plant community, and climatic (global only) predictors.

For the local analysis, we identified highly correlated variables using the variance inflation factor (VIF), dropped one of them, and repeated the process until no correlation was found. Then, we retained every predictor with P-value < 0.1. At the local scale, whole community descriptors were better predictors of AMF and pathogens. For saprotrophs, a model including legume biomass was similar to the best predictive model using only whole community plant descriptors. We opted for the whole community model because some sites did not have functional group information.

We applied a backwards stepwise model using the Bayesian Information Criterion (BIC) to fit the climatic and edaphic models for the global analysis. For the global regression model based on soil predictors, we included only the most significant predictors found in the local model. For plants, because of the small sample size for the global analysis, and because splitting the plant community into functional groups and using phylogenetic information increased the number of predictors, we decided to manually build the optimum fungal guild predictor models. We started fitting several regressions representing biomass and biodiversity

descriptors for each guild using whole community descriptors (total biomass, richness, PD, MPD, and MNTD, independently and in combination) and later replaced them with biomass and diversity metrics per functional group. We identified the best diversity descriptor for each functional group manually permuting among species richness, PD, MPD, and MNTD, and retained the most informative functional group diversity metric for each guild. In general, splitting the plant community by plant functional groups always provided a better or equivalent fit than treating the whole plant community as a unit at the global level (Supplementary Table 8).

Details of the Structural Equation Model analysis

Non-significant variables that were identified as the best predictors in each sub-group were also included in the SEM to test their independence. We added missing links either as causal connections or covariates when needed, and estimated the final Fisher's C and the standardized path coefficients. For each covariate included in the model, we tested the effect of treatment and, if significant, we added the corresponding paths in the SEM (Supplementary Table 1). We report the variation explained by the fixed factors only (Marginal R^2) as well as the total amount of variation explained by our mixed-effect models (Conditional R^2, Supplementary Table 9)[46]. Missing links that were marginally significant ($P<0.1$, Supplementary Table 10) were also included. Using a fixed effects model, we confirmed the lack of interaction between treatment and site (Supplementary Table 5). Finally, using a log-likelihood test we confirmed that the interaction between year and treatment was not significant ($P > 0.05$). Multi-year studies could shed light in the long-term effect of fertilization and provide stronger inference for causal analyses.

Mediation test of the global regressions

For each guild model fitted with climate variables, we tested if any of the predictors retained in the vegetation or climate models could add relevant information. We identified these other relevant predictors using the conditional independence test provided by the *dSep* function in the *piecewiseSEM* package[47]. We also included location variables (elevation, distance to

equator in degrees, hemisphere) to account for potential processes not included in the original set of variables but correlated with location. We expanded the original climate model adding any variable that was non-independent (p-value < 0.1) from the guild abundance after conditioning for the variables already included in the climate model. The same procedure was repeated for each guild starting with the soil and the plant models. We compared the expanded models with the original ones and identified the most parsimonious model for each guild. Because not all sites have detailed information for plant functional groups, we opted to keep the whole plant community descriptors for the SEM because we have little evidence that adding functional groups improved the models and many sites did not have that detailed information available. Finally, we run a step-backwards selection model to identify potential duplicity between the added predictors and the ones already included in the model. All the model comparisons were done using the Bayesian Information Criterion (BIC) as it penalizes more the overparameterization than the Akaike Information Criterion (AIC).

We estimated the relative explanatory power of each model as the R^2, and the variance explained by predictor type —climate, soil, biomass, biodiversity, location— as the difference between the R^2 of the full model minus the R^2 of the model without those variables. Plant community effect was estimated independently by dropping biomass and biodiversity variables, and also by dropping biomass and biodiversity variables together.

We tested the final models for residual normality using a Shapiro-Wilks test, visually explored residuals heteroskedasticity, and confirmed that outliers, when present, had no effect on the regressions (Supplementary Table 6). We found evidence of suppression effect (e.g., including plant community and location predictors explained more variability than including either set of variables independently), suggesting that simultaneous direct and indirect effects of opposing signs connect some variables and drive observed global trends.

Supplementary Tables

Supplementary Table 1. Effect of treatment on post-treatment predictor variables. Variables retained in the in the SEM are marked with †. Variable names according to Supplementary Table 7. Numbers in bracket indicate the p-value. Predictors' significance test based on the two-sided t-statistic of the estimated mean effect. Regression descriptors include the standard deviation the error terms (residual and site_code), the marginal and conditional R^2, the p-value of the normality test of both error terms (residual and site_code), and the number of groups and of observations.

Soil properties

**Predictor**	**dpH†**	**lRootsgperm2**
(Intercept)	0.383 (<0.001)	5.599 (<0.001)
TreatmentN	-0.184 (0.007)	-0.138 (0.128)
TreatmentNP	-0.074 (0.277)	0.023 (0.801)
TreatmentP	-0.062 (0.369)	-0.008 (0.932)
**Model descriptors**		
sd_Residual	0.372	0.605
sd_site_code	0.316	0.948
Marginal	0.0181	0.00311
Conditional	0.4301	0.7116
Shapiro(p.value) (site_code)	0.3635	0.462
Shapiro(p.value) (residuals)	1.14E-02	8.69E-02
Groups site_code	21	29
Observations	242	358

Litter and plant biomass

**Predictor**	**litter_mass**	**llive_mass†**	**lgram_mass**	**llegu_mass**	**lforb_mass**
(Intercept)	3.402 (<0.001)	5.544 (<0.001)	5.069 (<0.001)	1.008 (<0.001)	3.401 (<0.001)
TreatmentN	-0.057 (0.613)	0.183 (0.013)	-0.126 (0.326)	-0.239 (0.121)	0.238 (0.202)
TreatmentNP	0.058 (0.604)	0.359 (<0.001)	0.134 (0.293)	-0.304 (0.049)	0.492 (0.008)
TreatmentP	0.067 (0.549)	0.225 (0.002)	-0.035 (0.786)	0.275 (0.075)	0.339 (0.069)
**Model descriptors**					
sd_Residual	0.717	0.469	0.818	0.986	1.194
sd_site_code	2.464	0.84	1.004	1.194	1.436
Marginal	0.0003732	0.01778	0.005145	0.02102	0.009255
Conditional	0.922	0.7664	0.6028	0.6032	0.5951
Shapiro(p.value) (site_code)	3.98E-03	0.698	5.67E-01	6.66E-05	8.95E-01
Shapiro(p.value) (residuals)	2.85E-15	2.44E-04	2.93E-14	8.43E-14	9.21E-06
Groups site_code	26	26	26	26	26
Observations	328	328	328	328	328

Plant community richness

Predictor	sr	sr_gr	sr_lg	sr_fr
(Intercept)	11.852 (<0.001)	4.421 (<0.001)	1.036 (<0.001)	6.396 (<0.001)
TreatmentN	-0.044 (0.906)	-0.005 (0.976)	-0.236 (0.006)	0.195 (0.528)
TreatmentNP	-0.496 (0.182)	-0.119 (0.445)	-0.227 (0.008)	-0.152 (0.623)
TreatmentP	-0.156 (0.674)	-0.117 (0.454)	0.034 (0.692)	-0.075 (0.809)
**Model descriptors**				
sd_Residual	2.494	1.050	0.572	2.074
sd_site_code	6.360	2.524	0.925	4.002
Marginal	8.14E-04	4.50E-04	1.30E-02	8.12E-04
Conditional	0.867	0.853	0.727	0.789
Shapiro(p.value) (site_code)	1.38E-03	2.71E-03	2.47E-03	1.16E-02
Shapiro(p.value) (residuals)	2.10E-06	1.52E-02	8.35E-11	2.02E-07
Groups site_code	29	29	29	29
Observations	360	360	360	360

Plant community Faith's PD

Predictor	pd	pd_gr	pd_lg	pd_fr
(Intercept)	1175.657 (<0.001)	481.394 (<0.001)	291.080 (<0.001)	871.840 (<0.001)
TreatmentN	-5.202 (0.844)	4.236 (0.665)	-46.388 (0.025)	6.159 (0.828)
TreatmentNP	-21.936 (0.406)	-9.754 (0.317)	-66.297 (0.001)	-15.791 (0.576)
TreatmentP	3.850 (0.884)	4.581 (0.639)	-17.137 (0.407)	5.342 (0.850)
**Model descriptors**				
sd_Residual	177.672	65.586	138.698	189.753
sd_site_code	343.337	93.112	187.172	326.685
Marginal	6.49E-04	2.57E-03	1.21E-02	5.45E-04
Conditional	0.789	0.669	0.650	0.748
Shapiro(p.value) (site_code)	6.19E-02	2.53E-04	2.02E-03	1.50E-01
Shapiro(p.value) (residuals)	9.55E-03	3.70E-28	2.79E-11	2.42E-04
Groups site_code	29	29	29	29
Observations	360	360	360	360

Plant community Faith's MPD

Predictor	mpd	mpd_gr	mpd_fr
(Intercept)	267.312 (<0.001)	46.454 (<0.001)	212.094 (<0.001)
TreatmentN	-6.244 (0.224)	-1.351 (0.671)	-8.458 (0.373)
TreatmentNP	-3.852 (0.452)	-0.565 (0.860)	-13.872 (0.147)
TreatmentP	2.936 (0.567)	-4.027 (0.205)	-1.716 (0.857)
**Model descriptors**			
sd_Residual	34.419	21.002	62.750
sd_site_code	25.367	42.288	59.383
Marginal	6.71E-03	1.07E-03	4.08E-03
Conditional	0.356	0.802	0.475
Shapiro(p.value) (site_code)	1.62E-03	1.40E-03	1.49E-01
Shapiro(p.value) (residuals)	4.75E-20	3.46E-21	7.08E-09
Groups site_code	29	29	29
Observations	360	348	347

Plant community Faith's MNTD

Predictor	mntd†	mntd_gr	mntd_fr
(Intercept)	106.876 (<0.001)	26.715 (<0.001)	148.005 (<0.001)
TreatmentN	-8.736 (0.140)	-2.385 (0.463)	-9.746 (0.260)
TreatmentNP	-2.627 (0.655)	-0.916 (0.779)	-12.890 (0.139)
TreatmentP	-2.397 (0.685)	-5.152 (0.113)	-5.410 (0.533)
**Model descriptors**			
sd_Residual	39.619	21.490	57.184
sd_site_code	35.733	28.806	47.870
Marginal	3.65E-03	2.96E-03	4.23E-03
Conditional	0.451	0.644	0.415
Shapiro(p.value) (site_code)	2.05E-02	1.37E-05	5.01E-01
Shapiro(p.value) (residuals)	2.13E-17	1.52E-21	1.29E-05
Groups site_code	29	29	29
Observations	360	348	347

Plant community Jaccard's dissimilarity (percentage)

Predictor	All_jaccard†	gr_jaccard	lg_jaccard	fr_jaccard
(Intercept)	1.292 (0.589)	0.653 (0.821)	1.539 (0.680)	1.515 (0.625)
TreatmentN	49.498 (<0.001)	35.433 (<0.001)	17.455 (<0.001)	52.394 (<0.001)
TreatmentNP	51.413 (<0.001)	36.902 (<0.001)	15.854 (<0.001)	51.398 (<0.001)
TreatmentP	47.063 (<0.001)	35.130 (<0.001)	15.733 (<0.001)	49.514 (<0.001)
**Model descriptors**				
sd_Residual	12.05	19.3	21.919	17.418
sd_site_code	10.82	11.025	15.732	13.345
Marginal	0.6406	0.3321	0.06612	0.5095
Conditional	0.801	0.4965	0.3836	0.6909
Shapiro(p.value) (site_code)	0.2578	0.2193	0.0001008	0.76
Shapiro(p.value) (residuals)	0.7425	0.0009053	2.41E-17	0.001304
Groups site_code	29	29	29	29
Observations	359	359	359	359

Supplementary Table 2. Principal sources of variability as described by different models. Among site variability is captured by the difference between conditional and marginal variability. *Final* model includes all the terms presented in Supplementary Table 3. *Final & Treatment* models include the treatments to assess if treatment provides significant explanatory power to the final model. *Soil & Treatment* aims test if treatment is a better predictor than plant communities by replacing plant community predictors with treatment. *Plant community* only uses the plant community predictors to assess how much variability can be explained by the plant predictors alone that were measured at the end of the experiment. Finally, *Pre-treatment* only includes soil pre-treatment descriptors. Log-likelihood tests between the *Final* and *Final & Treatment* models confirmed that they are equivalent ($p > 0.2$).

**Guild**	**Model**	**df**	**Marginal**	**Conditional**
			Fixed terms	Fixed terms + random site term
AMF	Final	8	0.141	0.719
	Final & Treatment	11	0.136	0.712
	Soil & Treatment	9	0.095	0.681
	Plant community	5	0.036	0.656
	Pre-treatment	5	0.102	0.682
Pathogens	Final	7	0.305	0.426
	Final & Treatment	10	0.306	0.433
	Soil & Treatment	9	0.299	0.434
	Plant community	4	0.076	0.410
	Pre-treatment	5	0.213	0.342
Saprotrophs	Final	9	0.255	0.415
	Final & Treatment	12	0.260	0.438
	Soil & Treatment	10	0.190	0.458
	Plant community	5	0.124	0.393
	Pre-treatment	6	0.181	0.426

Supplementary Table 3. Local SEM model coefficients from the best fit models to explain local factors that shape fungal guilds. All models with random site as a random intercept. Shaded rows indicate differences among both final models. Variable names according to Supplementary Table 7. Significance test based on the two-sided t-statistic of the estimated mean effect: $^{(*)}$ signify P=0.1, * P<0.05, ** P<0.01, ***P<0.001.

Assuming that saprotrophs are correlated with pathogens and AMF, but not directly connected

Fisher's C = 151.2, df=150, p-value=0.457, AIC=263.05, K=56, n=159

Response	Predictor	Estimate	Std.Error	DF	Crit.Value	P.Value	Std.Estimate	
l_AMF	ppm_Fe	-0.0046	0.0016	96.0275	-2.8636	0.0051	-0.4386	**
l_AMF	dpH	0.2878	0.1751	149.9802	1.6436	0.1024	0.1207	
l_AMF	llive_mass	-0.2207	0.0984	152.9633	-2.2435	0.0263	-0.1835	*
l_AMF	mntd	-0.0027	0.0013	150.8287	-2.0409	0.043	-0.136	*
l_AMF	pH	-0.3422	0.204	99.3466	-1.6771	0.0967	-0.2584	
l_Pat	ppm_Mg_100	0.071	0.0332	20.8394	2.1402	0.0443	0.2531	*
l_Pat	dpH	-0.2751	0.1691	133.0663	-1.627	1.06E-01	-0.1445	
l_Pat	All_jaccardx100	0.0093	0.0023	151.6522	4.1276	1.00E-04	0.271	***
l_Pat	pH	0.2751	0.1316	30.8214	2.0898	0.045	0.2602	*
l_Sap	ppm_Mg_100	-0.0615	3.23E-02	18.8144	-1.9028	0.0725	-0.2539	
l_Sap	dpH	0.2657	0.1493	133.0979	1.7794	7.75E-02	0.1616	
l_Sap	lRootsgperm2	0.2206	7.60E-02	56.2662	2.9007	0.0053	0.2738	**
l_Sap	All_jaccardx100	0.0037	2.00E-03	146.7496	1.8937	0.0602	0.126	
l_Sap	pH	0.1168	0.135	28.524	0.8651	0.3942	0.1279	
l_Sap	ppm_Fe	0.0022	9.00E-04	20.0609	2.3474	0.0293	0.3035	*
~~l_Pat	~~l_Sap	-0.2458	-	159	-3.1667	9.00E-04	-0.2458	***
dpH	N1	-0.2863	6.53E-02	139.0256	-4.3852	0	-0.2666	***
dpH	P1	-0.0783	0.0675	139.0341	-1.16	2.48E-01	-0.0698	
dpH	NP	-0.1313	0.0667	138.9469	-1.9696	0.0509	-0.1193	
dpH	pH	-0.4064	0.0679	55.2573	-5.9875	0	-0.732	***
llive_mass	N1	0.2711	1.17E-01	141.0815	2.3133	0.0222	0.1272	*
llive_mass	P1	0.344	0.1212	141.1158	2.8384	0.0052	0.1547	**
llive_mass	NP	0.3763	0.1199	141.2006	3.1388	2.10E-03	0.1724	**
mntd	N1	-8.2103	9.1813	141.1885	-0.8942	0.3727	-0.0641	
mntd	P1	-2.0837	9.4955	141.2663	-0.2194	8.27E-01	-0.0156	
mntd	NP	-8.961	9.39E+00	141.4546	-0.954	0.3417	-0.0683	
All_jaccardx100	N1	50.2606	2.4228	141.1946	20.7451	0	0.8453	***
All_jaccardx100	P1	49.7103	2.5056	141.2843	19.8395	0	0.8013	***
All_jaccardx100	NP	52.5943	2.4783	141.5002	21.2217	0.00E+00	0.8633	***
lRootsgperm2	N1	-0.0629	0.1139	138.8288	-0.5523	0.5816	-0.0287	
lRootsgperm2	P1	0.0742	0.1178	138.9229	0.63	5.30E-01	0.0325	
lRootsgperm2	NP	-0.0666	0.117	139.344	-0.5693	0.5701	-0.0297	
lRootsgperm2	ppm_Fe	0.0042	0.0012	118.943	3.4954	7.00E-04	0.4628	***

Supplementary Table 4. Genera and guild affiliation of taxa that showed significant correlations in the co-occurrence analyses.

Treatments	Genera	Guild
Occurred in all treatments	*Archaeorhizomyces*	Saprotroph
	Clavaria	Saprotroph
	Coniella	Pathogen
	Embellisia	Pathogen
	Gibberella	Pathogen
	Glomus	AMF
	Hygrocybe	Saprotroph
	Lachnum	Saprotroph
	Lewia	Pathogen
	Mariannaea	Saprotroph
	Mortierella	Saprotroph
	Paecilomyces	Saprotroph
	Penicillium	Saprotroph
	Pyrenophora	Pathogen
	Thanatephorus	Pathogen
	Trichoderma	Saprotroph
	Ulocladium	Pathogen
	Umbelopsis	Saprotroph
Only in N, P, N+P soils	*Cylindrocarpon*	Pathogen
	Leptosphaeria	Pathogen
	Poculum	Saprotroph
Only in control soils	*Botryotinia*	Pathogen
	Clavulinopsis	Saprotroph
	Geoglossum	Saprotroph
	Gliomastix	Saprotroph
	Ramariopsis	Saprotroph
	Sesquicillium	Saprotroph

Supplementary Table 5. Test of the interaction of treatment and site, showing no interaction for all the fungi guilds. A significant interaction would prevent treating site as a random effect. Df = degrees of freedom, Sum sq = sums of squares, Mean sq = Mean square, F value = F statistic, Pr(>F) = p value. Variable names according to Supplementary Table 7.

Fungi guild/Factor	Df	Sum Sq	Mean Sq	F value	Pr(>F)
**Colonization**					
site_code	21	25.12	1.1964	2.20	0.0050
Treatment	3	1.55	0.5169	0.95	0.4187
site_code:Treatment	63	48.82	0.7750	1.43	0.0564
site_code:factor(block)	45	26.70	0.5934	1.09	0.3516
Residuals	98	53.21	0.5430		
**AMF**					
site_code	18	149.53	8.3070	19.73	0.0000
Treatment	3	2.96	0.9877	2.35	0.0783
llive_mass	1	0.66	0.6567	1.56	0.2151
ppm_Fe	1	0.54	0.5407	1.28	0.2602
ppm_K	1	3.60	3.6006	8.55	0.0044
site_code:Treatment	54	22.02	0.4078	0.97	0.5440
site_code:factor(block)	36	27.57	0.7657	1.82	0.0125
Residuals	87	36.63	0.4210		
**Pathogens**					
site_code	18	55.07	3.0594	6.43	0.0000
Treatment	3	11.55	3.8488	8.09	0.0001
dpH	1	2.08	2.0799	4.37	0.0403
ppm_Mg	1	1.39	1.3934	2.93	0.0916
site_code:Treatment	45	19.42	0.4315	0.91	0.6315
site_code:factor(block)	31	16.54	0.5335	1.12	0.3400
Residuals	67	31.86	0.4756		
**Saprotrophs**					
site_code	18	64.57	3.5874	15.81	0.0000
Treatment	3	0.59	0.1955	0.86	0.4656
dpH	1	1.52	1.5191	6.69	0.0119
lRootsgperm2	1	2.14	2.1366	9.42	0.0031
ppm_Fe	1	0.01	0.0088	0.04	0.8446
site_code:Treatment	45	15.37	0.3415	1.51	0.0643
site_code:factor(block)	31	21.66	0.6986	3.08	0.00006
Residuals	66	14.98	0.2269		

Supplementary Table 6. Coefficients and regression descriptors of the global models, including the standard deviation (sd) of the residuals, explanatory power (R^2), normality test of the residuals, and number of sites (observations) included in the model. All response variables were log(+1)-transformed. Predictor names according to Supplementary Table 7. Values in brackets indicate the p-value based on the two-sided t-statistic of the estimated mean effect.

**Predictor**	**Colonization**	**AMF**	**Pathogens**	**Saprotrophs**
(Intercept)	1.697 (0.014)	10.602 (<0.001)	-2.702 (0.076)	7.324 (<0.001)
elevation		-0.001 (<0.001)		
MAT_v2				-0.066 (0.015)
TEMP_VAR_v2				-0.003 (<0.001)
MAP_VAR_v2				-0.013 (0.023)
pH			0.883 (0.001)	
ppm_Fe	-0.003 (0.026)		0.004 (0.084)	
ppm_Mg				-0.001 (0.013)
lgram_mass		-0.634 (0.044)		
lforb_mass		-0.511 (0.020)		
llegu_mass	0.134 (0.033)			
lRootsgperm2	0.259 (0.015)			
SR_lg		-0.259 (0.005)		
mntd_fr		-0.010 (0.019)		
sd(residuals)	0.3792	0.5136	0.5647	0.4718
R^2	0.6082	0.8108	0.4994	0.7399
Shapiro test (p.value) (residuals)	0.6838	0.815	0.9237	0.3041
Observations	20	19	19	19

Supplementary Table 7. List of variables used.

A. Fungal guild variables

Original variable name	Definition	Variable name (log+1-transformed)
per_amf	Colonization of Root-AMF	lper_amf
N_AMF	Soil AMF	l_AMF
N_Pat	Soil pathogen	l_Pat
N_Sap	Soil saprotrophs	l_Sap

B. Plant community variables

	Biomass (log+1-transformed)	Richness	Faiht's Phylogenetic distance (PD)	Mean Phylogenetic Distance (MPD)	Mean Nearest Taxonomic Distance (MNTD)	Jaccard dissimilarity
Whole community	ltotal_mass	SR	PD	mpd	mntd	All_jaccard
Forbs	lforb_mass	SR_fr	PD_fr	mpd_fr	mntd_fr	fr_jaccard
Graminoids	lgram_mass	SR_gr	PD_gr	mpd_gr	mntd_gr	gr_jaccard
Legumes	llegu_mass	SR_lg	PD_lg	mpd_lg	mntd_lg	lg_jaccard
Litter	llitter_mass					

C. Post-treatment soil measurements

Definition	Variable name
pH	pH_rootsample
Root biomass	Rootsgperm2 (log+1-transformed)
Change in pH	dpH = pH_rootsample - pH

D. Pre-treatment soil properties

Variable name	Transformation	Definition
pH	pH	
pct_C	pct_C	Soil carbon concentration (% by mass)
pct_N	pct_N	Soil nitrogen concentration (% by mass)
ppm_P	ppm_P	Soil phosphorus concentration (ppm by mass)
ppm_K	ppm_K	Soil potassium concentration (ppm by mass)
ppm_Ca	ppm_Ca_100 = ppm_Ca/100	Soil calcium concentration (ppm by mass/100)
ppm_Mg	ppm_Mg_100 = ppm_Mg/100	Soil magnesium concentration (ppm by mass/100)
ppm_S	ppm_S	Soil sulphur concentration (ppm by mass)
ppm_Na	ppm_Na_10	Soil sodium concentration (ppm by mass)
ppm_Zn	ppm_Zn	Soil zinc concentration (ppm by mass)
ppm_Mn	ppm_Mn	Soil manganese concentration (ppm by mass)
ppm_Fe	ppm_Fe	Soil iron concentration (ppm by mass)
ppm_Cu	ppm_Cu	Soil copper concentration (ppm by mass)
ppm_B	ppm_B	Soil boron concentration (ppm by mass)
C_N	C_N = pct_C/pct_N	C:N ratio
N_P	N_P_x1000 = pct_N/ppm_P *1000	N:P ratio

E. Climatic and location drivers considered in the model indicating the variables kept after removing highly correlated ones.

Variable	Kept	Definition
RAIN_PET		Rainfall - potential evapotranspiration
MAT_v2	X	Annual Mean Temperature (BIOCLIM v2)
MAT_RANGE_v2	X	Mean Diurnal Range (Mean of monthly (max temp - min temp)) (BIOCLIM v2)
ISO_v2		Isothermality (Diurnal Range/Annual Range) (* 100) (BIOCLIM v2)
TEMP_VAR_v2	x	Temperature Seasonality (standard deviation *100) (BIOCLIM v2)
MAX_TEMP_v2		Max Temperature of Warmest Month (BIOCLIM v2)
MIN_TEMP_v2		Min Temperature of Coldest Month (BIOCLIM v2)
ANN_TEMP_RANGE_v2		Temperature Annual Range (BIOCLIM v2)
TEMP_WET_Q_v2		Mean Temperature of Wettest Quarter (BIOCLIM v2)
TEMP_DRY_Q_v2		Mean Temperature of Driest Quarter (BIOCLIM v2)
TEMP_WARM_Q_v2		Mean Temperature of Warmest Quarter (BIOCLIM v2)
TEMP_COLD_Q_v2		Mean Temperature of Coldest Quarter (BIOCLIM v2)
MAP_v2	x	Mean Annual Precipitation (BIOCLIM v2)
MAP_WET_M_v2		Precipitation of Wettest Month (BIOCLIM v2)
MAP_DRY_M_v2		Precipitation of Driest Month (BIOCLIM v2)
MAP_VAR_v2	x	Precipitation Seasonality (Coefficient of Variation) (BIOCLIM v2)
MAP_WET_Q_v2		Precipitation of Wettest Quarter (BIOCLIM v2)
MAP_DRY_Q_v2		Precipitation of Driest Quarter (BIOCLIM v2)
MAP_WARM_Q_v2	x	Precipitation of Warmest Quarter (BIOCLIM v2)
MAP_COLD_Q_v2	x	Precipitation of Coldest Quarter (BIOCLIM v2)
AI		Aridity index
PET		Potential Evapotranspiration

F. Treatment variables

Variable name	Definition
TreatmentN, N1	Nitrogen addition
TreatmentP, P1	Phosphorus addition
TreatmentNP, NP	Nitrogen and phosphorus addition

Supplementary Table 8. Selection of the optimum set of variables to predict fungal guild abundances as a function of plant community properties for the global model. We include models using descriptors by the whole community and by functional group. When needed, and to confirm manual findings, we use a step-backwards model and indicate the variables dropped. All response variables were log+1 transformed before fitting the regressions, and samples were kept consistent by dropping sites with incomplete data. df = degrees of freedom. For each model, we reported the number of degrees of freedom in the model, the Bayesian Information Criterion (BIC) and the change in the BIC (∆BIC) comparing the best model (lowest BIC) with the corresponding one. Variable names according to Supplementary Table 7.

**A.Global trends: AMF root colonization model**	**df**	**BIC**	**∆BIC**
Whole community models			
per_amf ~ lplant_mass + SR	4	51.9756	7.7753
per_amf ~ lplant_mass + PD + mpd + mntd	6	56.8320	12.6318
per_amf ~ lplant_mass + PD + SR + mpd + mntd	7	59.3419	15.1417
step(per_amf ~ lplant_mass + PD + SR + mpd + mntd)	2	46.1394	1.9392
Partitioning the community by functional group			
per_amf ~ llegu_mass + lgram_mass + lforb_mass + SR_gr + SR_lg + SR_fr	8	53.0727	8.8725
per_amf ~ llegu_mass + lgram_mass + lforb_mass + PD_gr + SR_lg + SR_fr	8	51.9622	7.7620
per_amf ~ llegu_mass + lgram_mass + lforb_mass + mpd_gr + SR_lg + SR_fr	8	52.5132	8.3130
per_amf ~ llegu_mass + lgram_mass + lforb_mass + mntd_gr + SR_lg + SR_fr	8	52.2579	8.0576
per_amf ~ llegu_mass + lgram_mass + lforb_mass + PD_gr + PD_lg + SR_fr	8	51.1867	6.9865
per_amf ~ llegu_mass + lgram_mass + lforb_mass + PD_gr + PD_lg + PD_fr	8	50.7744	6.5742
per_amf ~ llegu_mass + lgram_mass + lforb_mass + PD_gr + PD_lg + mpd_fr	8	52.8349	8.6347
per_amf ~ llegu_mass + lgram_mass + lforb_mass + PD_gr + PD_lg + mntd_fr	8	53.4435	9.2433
step(per_amf ~ llegu_mass + lgram_mass + lforb_mass + PD_gr + PD_lg + PD_fr)	4	44.2002	0.0000

**B. Global trends: AMF soil abundance model**	**df**	**BIC**	**∆BIC**
Whole community models			
l_AMF ~ lplant_mass + SR	4	72.6272	7.8924
l_AMF ~ lplant_mass + PD + mpd + mntd	6	77.1408	12.4059
l_AMF ~ lplant_mass + PD + SR + mpd + mntd	7	79.2930	14.5581
step(l_AMF ~ lplant_mass + PD + SR + mpd + mntd)	2	69.9855	5.2507
Partitioning the community by functional group			
l_AMF ~ llegu_mass + lgram_mass + lforb_mass	5	70.7865	6.0517
l_AMF ~ llegu_mass + lgram_mass + lforb_mass + SR_gr + SR_lg + SR_fr	8	72.9896	8.2548
l_AMF ~ llegu_mass + lgram_mass + lforb_mass + PD_gr + SR_lg + SR_fr	8	71.9943	7.2594
l_AMF ~ llegu_mass + lgram_mass + lforb_mass + mpd_gr + SR_lg + SR_fr	8	73.2974	8.5625
l_AMF ~ llegu_mass + lgram_mass + lforb_mass + mntd_gr + SR_lg + SR_fr	8	73.5917	8.8569
l_AMF ~ llegu_mass + lgram_mass + lforb_mass + PD_gr + PD_lg + SR_fr	8	78.6847	13.9499
l_AMF ~ llegu_mass + lgram_mass + lforb_mass + PD_gr + SR_lg + PD_fr	8	73.4405	8.7056
l_AMF ~ llegu_mass + lgram_mass + lforb_mass + PD_gr + SR_lg + mpd_fr	8	73.4307	8.6959
l_AMF ~ llegu_mass + lgram_mass + lforb_mass + PD_gr + SR_lg + mntd_fr	8	69.0282	4.2933
step(l_AMF ~ llegu_mass + lgram_mass + lforb_mass + PD_gr + SR_lg + mntd_fr)	6	64.7348	0.0000

C. Global trends: Pathogen abundance model	df	BIC	ΔBIC
Whole community models			
l_Pat ~ lplant_mass + SR	4	57.5340	10.5419
l_Pat ~ lplant_mass + PD + mpd + mntd	6	57.6524	10.6603
l_Pat ~ lplant_mass + PD + SR + mpd + mntd	7	60.1404	13.1483
step(l_Pat ~ lplant_mass + PD + SR + mpd + mntd)	4	52.3375	5.3454
Partitioning the community by functional group			
l_Pat ~ llegu_mass + lgram_mass + lforb_mass	5	57.8347	10.8426
l_Pat ~ llegu_mass + lgram_mass + lforb_mass + SR_gr + SR_lg + SR_fr	8	65.3126	18.3205
l_Pat ~ llegu_mass + lgram_mass + lforb_mass + PD_gr + SR_lg + SR_fr	8	57.6922	10.7001
l_Pat ~ llegu_mass + lgram_mass + lforb_mass + mpd_gr + SR_lg + SR_fr	8	57.1551	10.1630
l_Pat ~ llegu_mass + lgram_mass + lforb_mass + mntd_gr + SR_lg + SR_fr	8	60.8897	13.8976
l_Pat ~ llegu_mass + lgram_mass + lforb_mass + mpd_gr + PD_lg + SR_fr	8	53.8221	6.8300
l_Pat ~ llegu_mass + lgram_mass + lforb_mass + mpd_gr + PD_lg + PD_fr	8	53.5546	6.5625
l_Pat ~ llegu_mass + lgram_mass + lforb_mass + mpd_gr + PD_lg + mpd_fr	8	52.7644	5.7723
l_Pat ~ llegu_mass + lgram_mass + lforb_mass + mpd_gr + PD_lg + mntd_fr	8	53.3264	6.3343
step(l_Pat ~ llegu_mass + lgram_mass + lforb_mass + mpd_gr + PD_lg + mpd_fr)	5	46.9921	0.0000

D. Global trends: Saprotrophs abundance models	df	BIC	ΔBIC
Whole community models			
l_Sap ~ llitter_mass + lplant_mass + SR	5	62.0437	6.3988
l_Sap ~ llitter_mass + lplant_mass + PD + mpd + mntd	7	64.0940	8.4491
l_Sap ~ llitter_mass + lplant_mass + PD + SR + mpd + mntd	8	67.1422	11.4973
step(l_Sap ~ llitter_mass + lplant_mass + PD + SR + mpd + mntd)	4	55.6449	0.0000
Partitioning the community by functional group			
l_Sap ~ llitter_mass + llegu_mass + lgram_mass + lforb_mass	6	63.3160	7.6711
l_Sap ~ llitter_mass + llegu_mass + lgram_mass + lforb_mass + SR_gr + SR_lg + SR_fr	9	70.1209	14.4760
l_Sap ~ llitter_mass + llegu_mass + lgram_mass + lforb_mass + PD_gr + SR_lg + SR_fr	9	69.4766	13.8317
l_Sap ~ llitter_mass + llegu_mass + lgram_mass + lforb_mass + mpd_gr + SR_lg + SR_fr	9	69.9876	14.3426
l_Sap ~ llitter_mass + llegu_mass + lgram_mass + lforb_mass + mntd_gr + SR_lg + SR_fr	9	70.6785	15.0336
l_Sap ~ llitter_mass + llegu_mass + lgram_mass + lforb_mass + PD_gr + PD_lg + SR_fr	9	66.5881	10.9431
l_Sap ~ llitter_mass + llegu_mass + lgram_mass + lforb_mass + PD_gr + PD_lg + PD_fr	9	66.2682	10.6233
l_Sap ~ llitter_mass + llegu_mass + lgram_mass + lforb_mass + PD_gr + PD_lg + mpd_fr	9	64.1836	8.5387
l_Sap ~ llitter_mass + llegu_mass + lgram_mass + lforb_mass + PD_gr + PD_lg + mntd_fr	9	61.3608	5.7159
step(l_Sap ~ llitter_mass + llegu_mass + lgram_mass + lforb_mass + PD_gr + PD_lg + mntd_fr)	7	56.8663	1.2214†

Note: †The model based on functional group was almost identical to the best model, which was based on whole community descriptors. We choose the former one to keep consistency with the other global models.

Supplementary Table 9. Descriptors of each regression included in the local SEM, including the standard deviation (sd) of the residuals and of the random intercepts (site), explanatory power as fixed effects only (Marginal R^2) or Mixed effects & Random effects (Conditional R^2), normality test of the residuals and the site intercepts, and number of sites and observations included in the model. All response variables, except change in pH (dpH) were log(+1) transformed. All models with random site as a random intercept. Model assuming that saprotrophs are correlated with pathogens and AMF, but not directly connected.

Model descriptor	AMF	Pathogens	Saprotrophs	Alive biomass	dpH	Roots biomass	Jaccard	Root biomass
sd(Residuals)	0.674	0.695	0.602	0.296	0.533	41.75	11.017	0.518
sd(Sites)	0.967	0.32	0.315	0.319	0.783	39.498	9.63	0.961
Marginal R^2	0.1407	0.305	0.255	0.4031	0.02427	0.0045	0.6999	0.1453
Conditional R^2	0.7189	0.4263	0.4146	0.724	0.6913	0.4747	0.8299	0.8079
Normality test (Shapiro-Wilk, p-value)								
- Sites	0.2283	0.5982	0.6019	0.6051	0.7718	0.4125	0.7233	0.15
- Residuals	0.6743	0.00016	0.8949	0.0007	0.1557	<0.001	0.7687	0.0260
Number of								
… sites	15	15	15	15	15	15	15	15
… observations	159	159	159	159	159	159	159	159

Supplementary Table 10. Local SEM performance: Missing links from the best-fit local SEM models ($p < 0.1$). Variable names according to Supplementary Table 7. Significance test based on the two-sided t-statistic of the estimated mean effect.

Independence claim	DF	Crit.Value	P.Value
**Assuming that saprotrophs are correlated with pathogens and AMF, but not directly connected**			
All_jaccardx100 ~ ppm_Fe + ...	31.11	3.054	0.090
lRootsgperm2 ~ ppm_Mg_100 + ...	56.66	3.139	0.082
l_AMF ~ ppm_Cu + ...	151.64	3.610	0.059
l_Pat ~ ppm_Mn + ...	18.24	3.507	0.077
lRootsgperm2 ~ All_jaccardx100 + ...	145.25	4.282	0.040
l_Sap ~ l_AMF + ...	65.99	3.168	0.080

Supplementary Figures

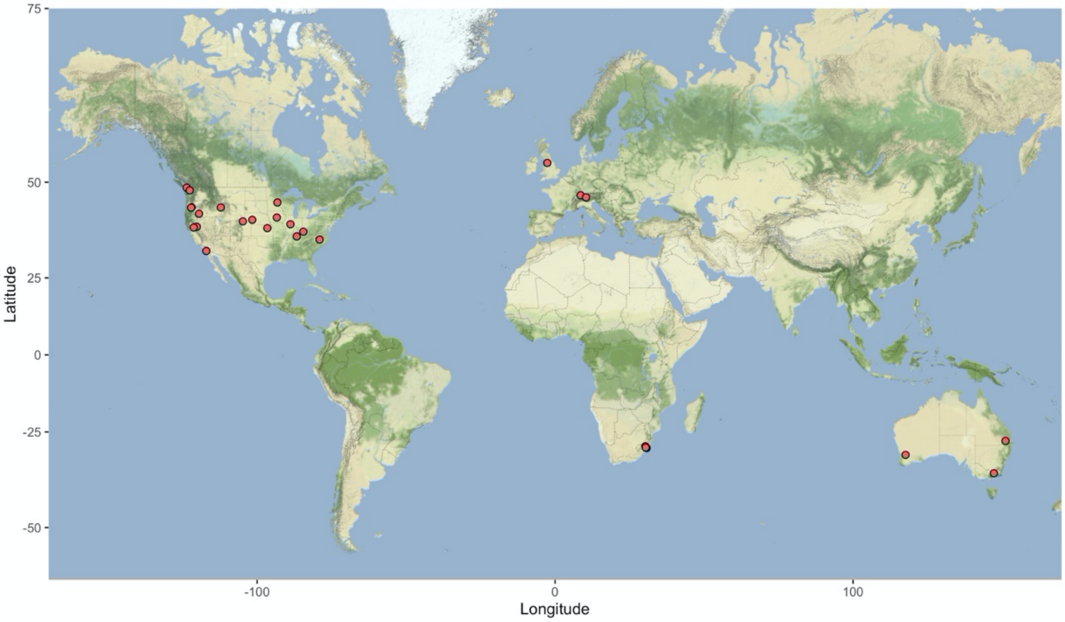

Supplementary Figure 1. Map of the 25 NutNet sites included in the study. The map was made using gg map[48].

a) AMF root colonization (percent)

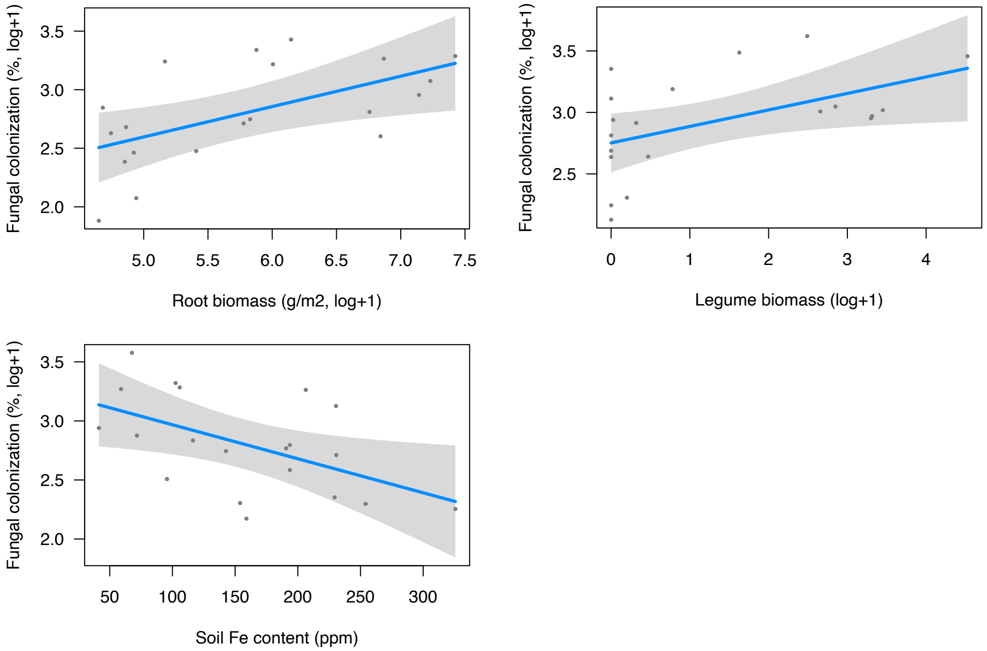

b) AMF soil colonization (sequence numbers)

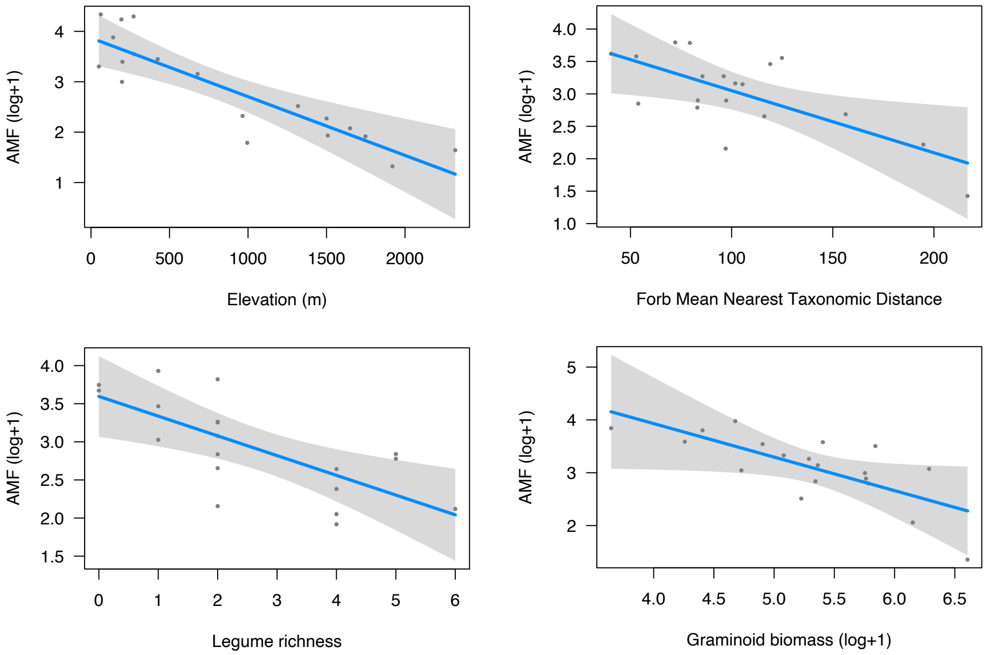

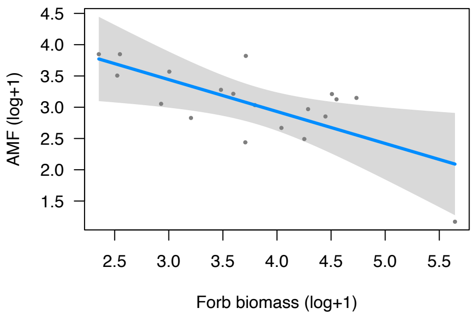

c) Soil pathogen abundance (sequence numbers)

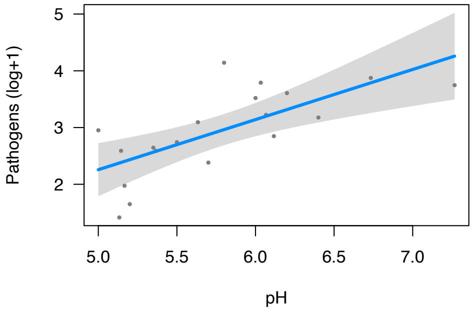

d) Soil saprotroph abundance (sequence numbers)

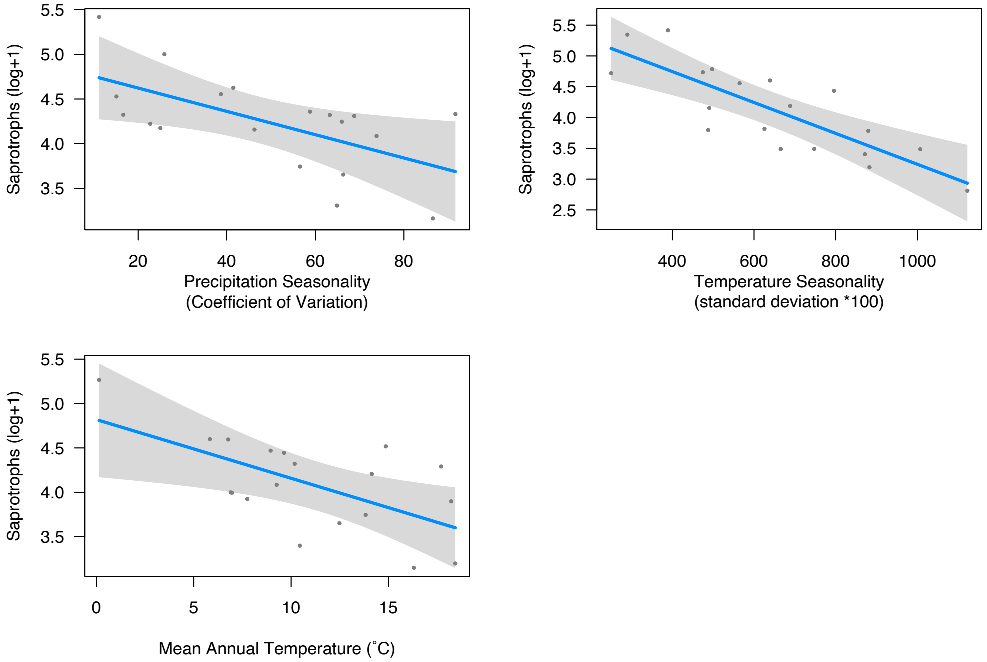

Supplementary Figure 2. Relationships between significant soil, plant, and climate parameters and arbuscular mycorrhizal fungal root (a) and soil colonization (b), pathogen abundance (c) and saprotroph abundance (d) across control plots (See Supplementary Table 6). The gray area represents the 0.95 confidence interval.

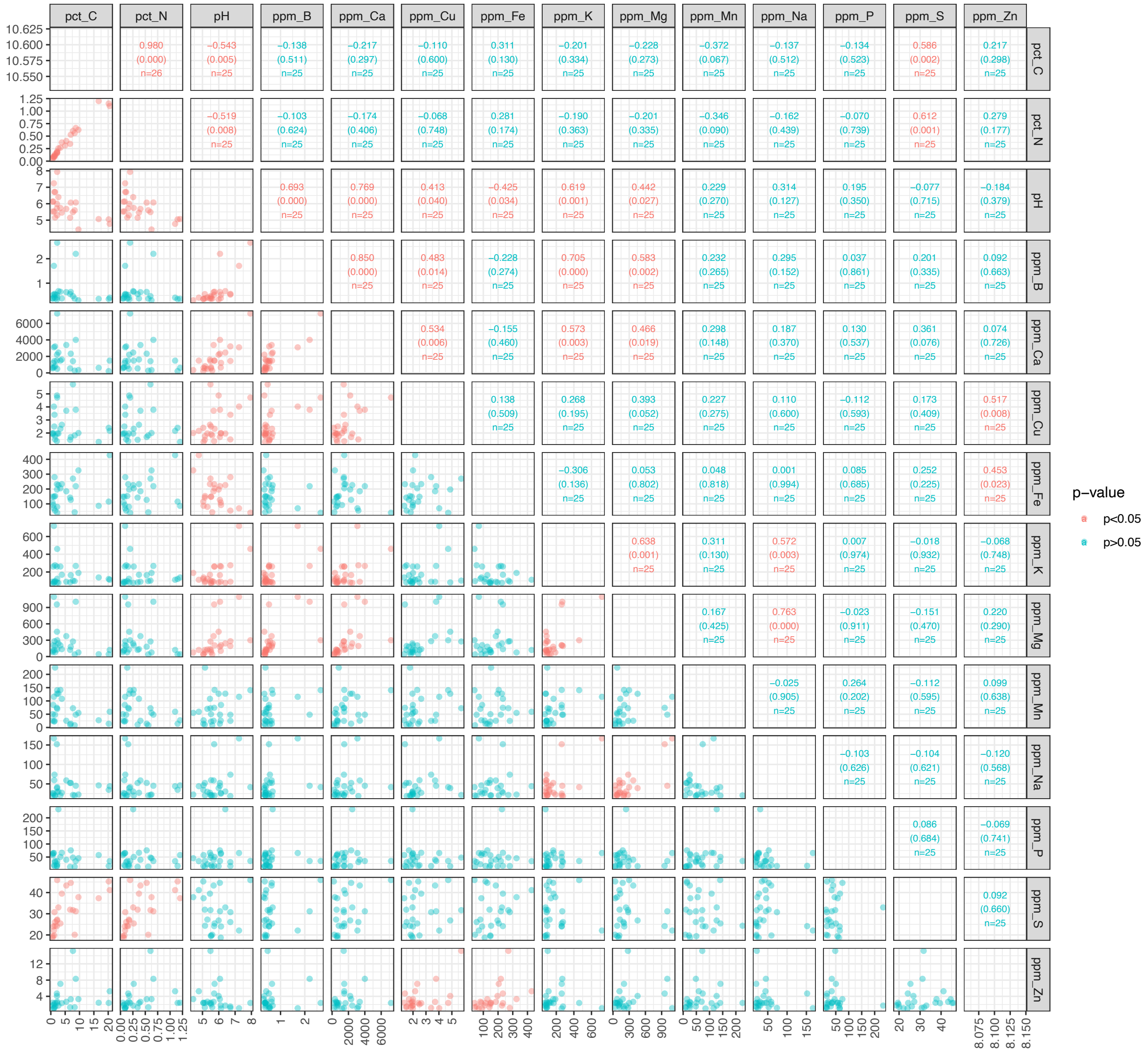

Supplementary Figure 3. Relationship between pre-treatment mean plot soil properties observed at each site. The lower left triangle shows the scatter plots of each of the corresponding pairs of variables while the upper triangle indicates the Pearson's correlation coefficient, the two-sided p-value (in brackets) and the number of observations. A panel with red dots indicates a significant correlation ($p < 0.05$), while blue dots indicate non-significant correlation between that pair of variables. Each dot is the average of the conditions observed in a given site before the treatments were applied.

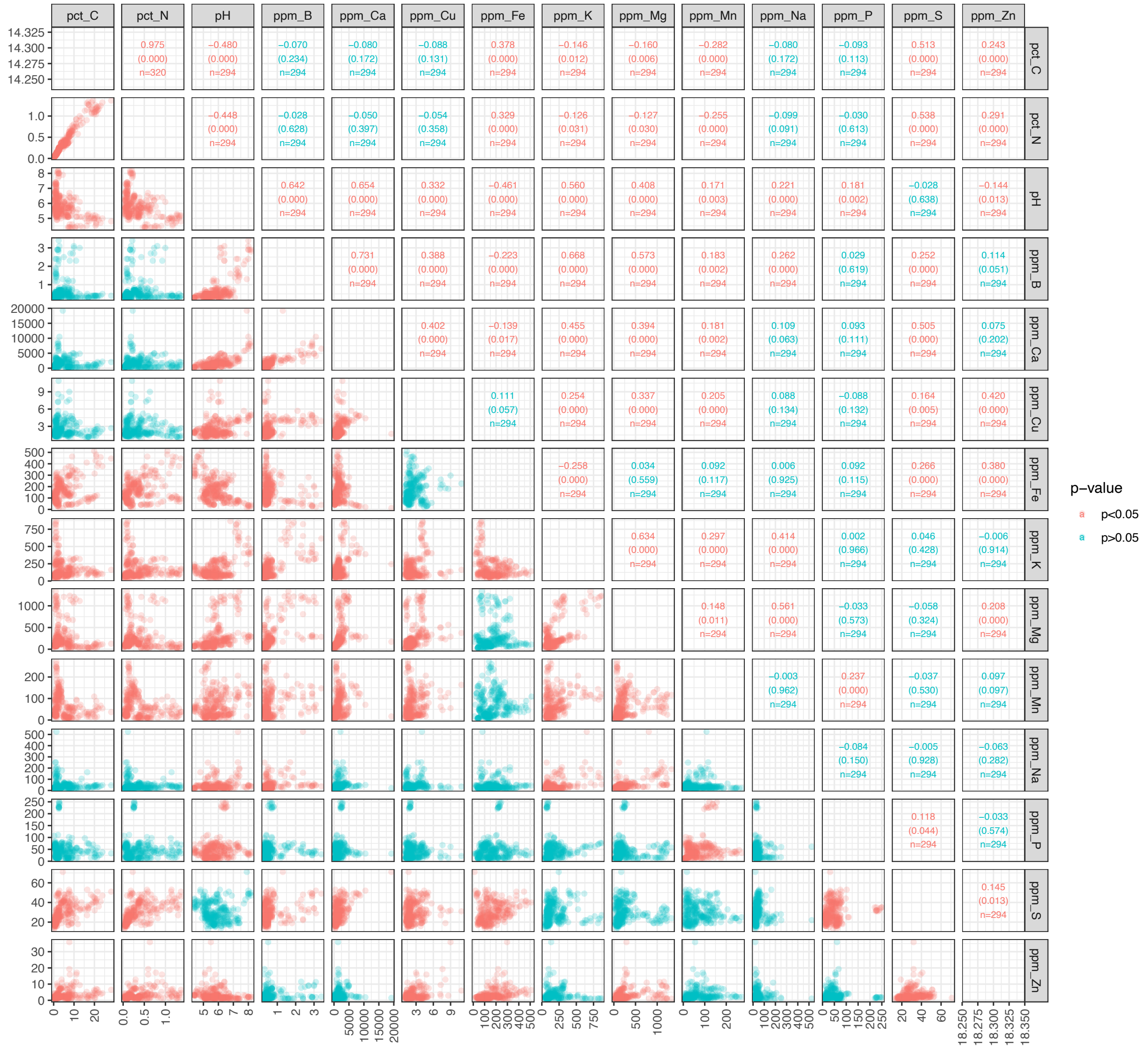

Supplementary Figure 4. Relationship between pre-treatment soil properties. The lower left triangle shows the scatter plots of each of the corresponding pairs of variables while the upper triangle indicates the Pearson's correlation coefficient, the two-sided p-value (in brackets) and the number of observations. A panel with red dots indicates a significant correlation ($p < 0.05$), while blue dots indicate non-significant correlation between that pair of variables. Each dot represents an individual plot before treatments.

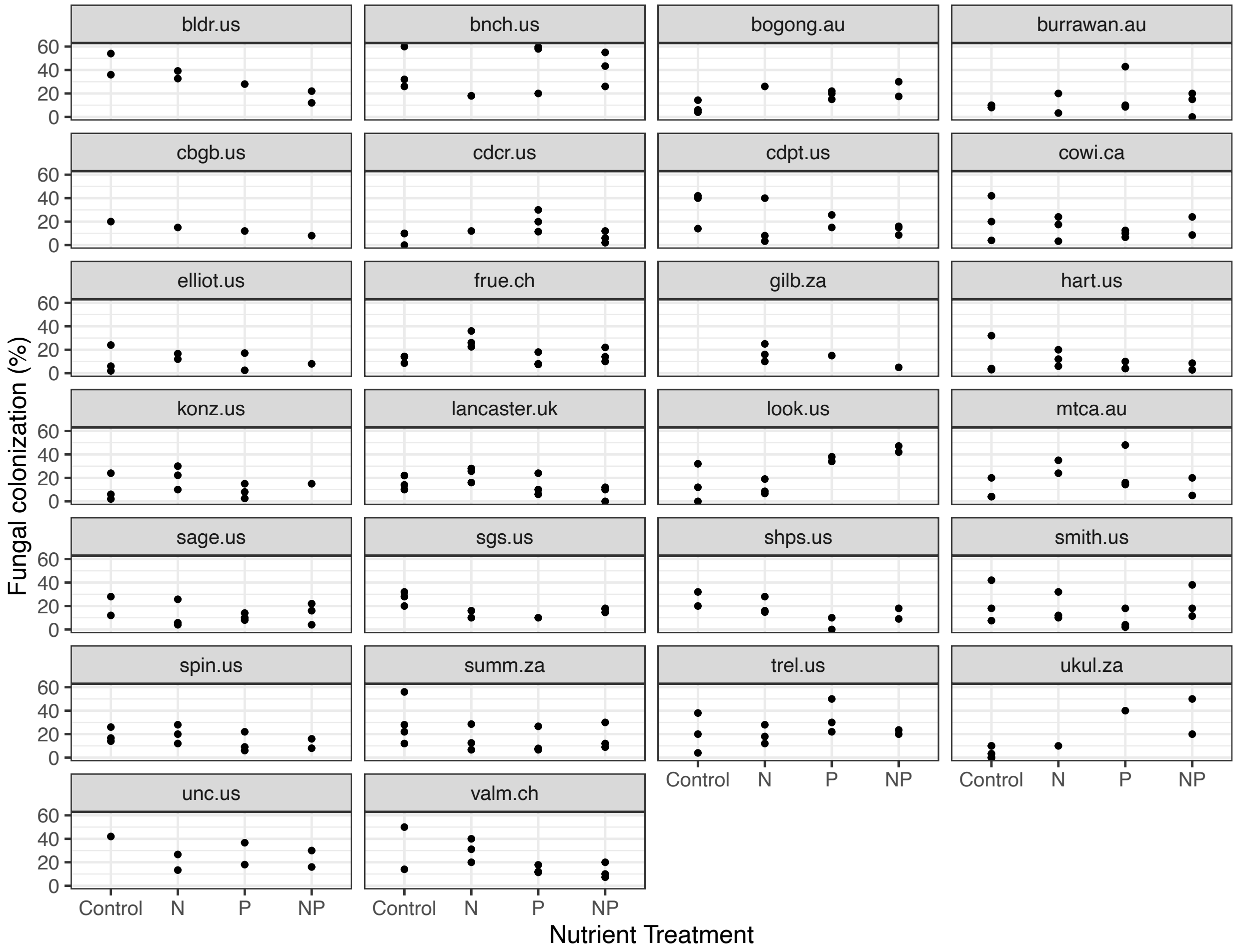

Supplementary Figure 5. Arbuscular mycorrhizal colonization (percent) response to nutrient addition at 26 grasslands. Three sites (hall.us, saline.us, sier.us) were removed from the figure because of low replicates across nutrient addition treatments. Black dots represent the values from individual plots.

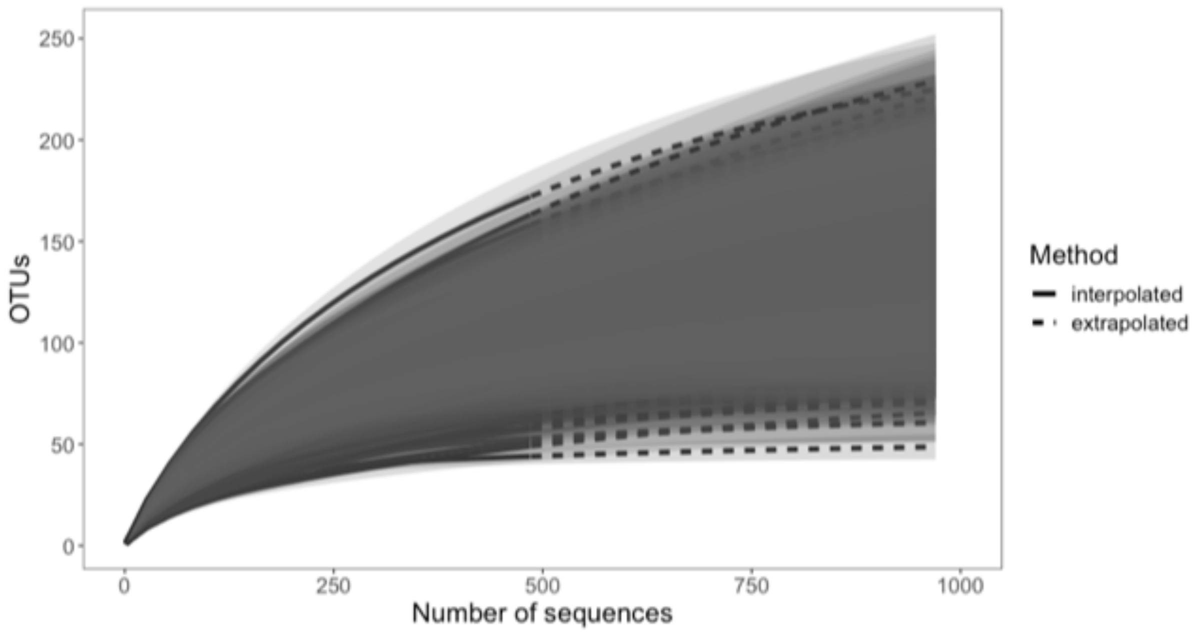

Supplementary Figure 6. Rarefaction curves of all 340 samples collected across all sites and treatments. While the rarefaction at 485 sequences did not allow for an exhaustive characterization of all taxa within all sites, it did most likely capture abundant taxa across sites.

Supplementary References

1. Johnson, N. C., Graham, J. H. & Smith, F. A. Functioning of mycorrhizal associations along the mutualism – parasitism continuum. *New Phytol.* 575–585 (1997). doi:10.1046/j.1469-8137.1997.00729.x
2. Delavaux, C. S., Smith-Ramesh, L. M. & Kuebbing, S. E. Beyond nutrients: a meta-analysis of the diverse effects of arbuscular mycorrhizal fungi on plants and soils. *Ecology* **98**, 2111–2119 (2017).
3. Smith, S. E. & Read, D. J. *Mycorrhizal Symbiosis*. (Academic Press, 2008).
4. Johnson, N. C. Resource stoichiometry elucidates the structure and function of arbuscular mycorrhizas across scales. *New Phytol.* **185**, 631–647 (2010).
5. Avolio, M. L. *et al.* Changes in plant community composition, not diversity, during a decade of nitrogen and phosphorus additions drive above-ground productivity in a tallgrass prairie. *J. Ecol.* **102**, 1649–1660 (2014).
6. Hartnett, D. C. & Wilson, G. W. T. Mycorrhizae Influence Plant Community Structure and Diversity in Tallgrass Prairie. *Ecology* **80**, 1187–1195 (1999).
7. Read, D. J. Mycorrhizas in ecosystems. *Experientia* **47**, 376–391 (1991).
8. Johnson, N. C., Wilson, G. W. T., Wilson, J. A., Miller, R. M. & Bowker, M. A. Mycorrhizal phenotypes and the Law of the Minimum. *New Phytol.* **205**, 1473–1484 (2015).
9. Kytöviita, M.-M. Asymmetric symbiont adaptation to Arctic conditions could explain why high Arctic plants are non-mycorrhizal. *FEMS Microbiol. Ecol.* **53**, 27–32 (2005).
10. Tedersoo, L. *et al.* Global diversity and geography of soil fungi. *Science (80-.).* **346**, 1052–1053 (2014).
11. Augé, R. M. Water relations , drought and vesicular-arbuscular mycorrhizal symbiosis. *Mycorrhiza* **11**, 3–42 (2001).
12. Wang, G. M., Stribley, D. P., Tinker, P. B. & Walker, C. Effects of pH on arbuscular mycorrhiza I . Field observations on the long-term liming experiments at Rothamsted and Woburn. *New Phytol.* **124**, 465–472 (1993).
13. Coughlan, A. P., Dalpé, Y., Lapointe, L. & Piché, Y. Soil pH-induced changes in root colonization , diversity , and reproduction of symbiotic arbuscular mycorrhizal fungi from

healthy and declining maple forests. *Can. J. For. Res.* **30**, 1543–1554 (2000).

14. Deyn, G. B. De, Quirk, H. & Bardgett, R. D. Plant species richness , identity and productivity differentially influence key groups of microbes in grassland soils of contrasting fertility. *Biol. Lett.* **7**, 75–78 (2011).

15. Wilson, G. W. T. & Hartnett, D. C. Interspecific variation in plant responses to mycorrhizal colonization in tallgrass prairie. *Am. J. Bot.* **85**, 1732–1738 (1998).

16. Hoeksema, J. D. *et al.* A meta-analysis of context-dependency in plant response to inoculation with mycorrhizal fungi. *Ecol. Lett.* **13**, 394–407 (2010).

17. Velásquez, A. C., Castroverde, C. D. M. & He, S. Y. Plant–Pathogen Warfare under Changing Climate Conditions. *Current Biology* **28**, R619–R634 (2018).

18. Mangan, S. A. *et al.* Negative plant–soil feedback predicts tree-species relative abundance in a tropical forest. *Nature* **466**, 752–755 (2010).

19. Maron, J. L., Marler, M., Klironomos, J. N. & Cleveland, C. C. Soil fungal pathogens and the relationship between plant diversity and productivity. *Ecol. Lett.* **14**, 36–41 (2011).

20. Klironomos, J. N. Feedback with soil biota contributes to plant rarity and invasiveness in communities. *Nature* **417**, 67–69 (2002).

21. Revillini, D., Gehring, C. A. & Johnson, N. C. The role of locally adapted mycorrhizas and rhizobacteria in plant–soil feedback systems. *Funct. Ecol.* **30**, 1086–1098 (2016).

22. Reynolds, H. L., Packer, A., Bever, J. D. & Clay, K. Grassroots Ecology : Plant-Microbe-Soil Interactions as Drivers of Plant Community Structure and Dynamics. *Ecology* **84**, 2281–2291 (2003).

23. Hersh, M. H. M. H., Vilgalys, R. R. & Clark, J. S. Evaluating the impacts of multiple generalist fungal pathogens on temperate tree seedling survival. *Ecology* **93**, 511–520 (2012).

24. Walters, D. R. & Bingham, I. J. Influence of nutrition on disease development caused by fungal pathogens: Implications for plant disease control. *Annals of Applied Biology* **151**, 307–324 (2007).

25. Veresoglou, S. D. & Rillig, M. C. Suppression of fungal and nematode plant pathogens through arbuscular mycorrhizal fungi. *Biol. Lett.* **8**, 214–217 (2012).

26. Givnish, T. J. On the causes of gradients in tropical tree diversity. *J. Ecol.* **87**, 193–210 (1999).
27. Schoeneweiss, D. F. Water stress predisposition to disease - an overview. in *Water, Fungi and Plants: Symposium of the British Mycologial Society* 157–174 (1985).
28. Packer, A. & Clay, K. Soil pathogens and spatial patterns of seedling mortality in a temperate tree. *Nature* **404**, 278–81 (2000).
29. Petermann, J. S., Fergus, A. J. F., Turnbull, L. A. & Schmid, B. Janzen-connell effects are widespread and strong enough to maintain diversity in grasslands. *Ecology* **89**, 2399–2406 (2008).
30. Knorr, M., Frey, S. D. & Curtis, P. S. Nitrogen additions and litter decomposition: A meta-analysis. *Ecology* **86**, 3252–3257 (2005).
31. Fogg, K. The effect of added nitrogen on the rate of decomposition of organic matter. *Biol. Rev.* **63**, 433–462 (1988).
32. Bonner, M. T. *et al.* Why does nitrogen addition to forest soils inhibit decomposition? *Soil Biol. Biochem.* **137**, 107570 (2019).
33. Zak, D. R. *et al.* Anthropogenic N deposition, fungal gene expression, and an increasing soil carbon sink in the Northern Hemisphere. *Ecology* **100**, 1–8 (2019).
34. Hobbie, S. E. *et al.* Response of decomposing litter and its microbial community to multiple forms of nitrogen enrichment. *Ecol. Monogr.* **82**, 389–405 (2012).
35. Van Der Heijden, M. G. A., Bardgett, R. D. & Van Straalen, N. M. The unseen majority: Soil microbes as drivers of plant diversity and productivity in terrestrial ecosystems. *Ecology Letters* **11**, 296–310 (2008).
36. Cornwell, W. K. *et al.* Plant species traits are the predominant control on litter decomposition rates within biomes worldwide. *Ecol. Lett.* **11**, 1065–1071 (2008).
37. Rousk, J. Growth of saprotrophic fungi and bacteria in soil ˚. (2011). doi:10.1111/j.1574-6941.2011.01106.x
38. Rousk, J., Brookes, P. C. & Bååth, E. Contrasting Soil pH Effects on Fungal and Bacterial Growth Suggest Functional Redundancy in Carbon Mineralization □ †. *Appl. Environ. Microbiol.* **75**, 1589–1596 (2009).

39. Borer, E. T. *et al.* Finding generality in ecology: A model for globally distributed experiments. *Methods Ecol. Evol.* **5**, 65–73 (2014).
40. Borer, E. T., Grace, J. B., Harpole, W. S., MacDougall, A. S. & Seabloom, E. W. A decade of insights into grassland ecosystem responses to global environmental change. *Nat. Ecol. Evol.* **1**, 0118 (2017).
41. Leff, J. W. *et al.* Consistent responses of soil microbial communities to elevated nutrient inputs in grasslands across the globe. *Proc. Natl. Acad. Sci.* **112**, 10967–10972 (2015).
42. Cleland, E. E. *et al.* Belowground Biomass Response to Nutrient Enrichment Depends on Light Limitation Across Globally Distributed Grasslands. *Ecosystems* **22**, 1466–1477 (2019).
43. McGonigle, T. P., Miller, M. H., Evans, D. G., Fairchild, G. L. & Swan, J. A. A new method which gives an objective measure of colonization of roots by vesicular—arbuscular mycorrhizal fungi. *New Phytol.* **115**, 495–501 (1990).
44. Fick, S. E. & Hijmans, R. J. WorldClim 2 : new 1-km spatial resolution climate surfaces for global land areas. *Int. J. Climatol.* **4315**, 4302–4315 (2017).
45. Faith, D. P. Conservation evaluation and phylogenetic diversity. *Biol. Conserv.* **61**, 1–10 (1992).
46. Nakagawa, S. & Schielzeth, H. A general and simple method for obtaining R2 from generalized linear mixed-effects models. *Methods Ecol. Evol.* **2**, 133–142 (2013).
47. Lefcheck, J. S. piecewiseSEM: Piecewise structural equation modelling in r for ecology, evolution, and systematics. *Methods Ecol. Evol.* **7**, 573–579 (2016).
48. Kahle, D. & Wickham, H. ggmap: Spatial visualization with ggplot 2. *R J.* **5**, 144–161 (2013).
