# Supplementary material for: Nitrogen and phosphorus fertilization consistently favor pathogenic over mutualistic fungi in grassland soils
Source: Nat Commun. 2021 Jun 9;12:3484. doi: 10.1038/s41467-021-23605-y (PMC8190096; doi:10.1038/s41467-021-23605-y)
Supplement: Supplementary file 3 — Reporting Summary [file 41467_2021_23605_MOESM3_ESM.pdf]

nature research

Corresponding author(s): Ylva Lekberg (ylekberg@mpgranch.com)

Last updated by author(s): Apr 28, 2021

# Reporting Summary

Nature Research wishes to improve the reproducibility of the work that we publish. This form provides structure for consistency and transparency in reporting. For further information on Nature Research policies, see our Editorial Policies and the Editorial Policy Checklist.

## Statistics

For all statistical analyses, confirm that the following items are present in the figure legend, table legend, main text, or Methods section.

| n/a | Confirmed | |
|---|---|---|
| ☐ | ☒ | The exact sample size (*n*) for each experimental group/condition, given as a discrete number and unit of measurement |
| ☐ | ☒ | A statement on whether measurements were taken from distinct samples or whether the same sample was measured repeatedly |
| ☐ | ☒ | The statistical test(s) used AND whether they are one- or two-sided<br>*Only common tests should be described solely by name; describe more complex techniques in the Methods section.* |
| ☐ | ☒ | A description of all covariates tested |
| ☐ | ☒ | A description of any assumptions or corrections, such as tests of normality and adjustment for multiple comparisons |
| ☐ | ☒ | A full description of the statistical parameters including central tendency (e.g. means) or other basic estimates (e.g. regression coefficient) AND variation (e.g. standard deviation) or associated estimates of uncertainty (e.g. confidence intervals) |
| ☐ | ☒ | For null hypothesis testing, the test statistic (e.g. *F*, *t*, *r*) with confidence intervals, effect sizes, degrees of freedom and *P* value noted<br>*Give P values as exact values whenever suitable.* |
| ☐ | ☒ | For Bayesian analysis, information on the choice of priors and Markov chain Monte Carlo settings |
| ☐ | ☒ | For hierarchical and complex designs, identification of the appropriate level for tests and full reporting of outcomes |
| ☐ | ☒ | Estimates of effect sizes (e.g. Cohen's *d*, Pearson's *r*), indicating how they were calculated |

*Our web collection on statistics for biologists contains articles on many of the points above.*

## Software and code

Policy information about availability of computer code

| | |
|---|---|
| Data collection | Fungal OTU data were matched to FUNGuild database (Nguyen NH, Song Z, Bates ST, Branco, S, Tedersoo L, Menke J, Schilling JS, Kennedy PG. 2016. FUNGuild: an open annotation tool for parsing fungal community datasets by ecological guild. Fungal Ecology 20:241-248.) to group fungal OTUs into functional groupings. |
| Data analysis | Source code and R scripts are available with EDI submission (https://doi.org/10.6073/pasta/f11f2fcf0e56978ce6d96b8b78dcd0d3). For the analyses we conducted, we performed them using R (version 4.0.3), RStudio (version 1.4.1106 ), with lmerTest (version 3.1-3), visreg (version 2.7.0), piecewiseSEM (version 2.1.2), SpiecEasi (version 1.1.1), vegan (version 2.5-7), ggplot2 (version 3.3.3) packages. |

For manuscripts utilizing custom algorithms or software that are central to the research but not yet described in published literature, software must be made available to editors and reviewers. We strongly encourage code deposition in a community repository (e.g. GitHub). See the Nature Research guidelines for submitting code & software for further information.

## Data

Policy information about availability of data

All manuscripts must include a data availability statement. This statement should provide the following information, where applicable:

- Accession codes, unique identifiers, or web links for publicly available datasets
- A list of figures that have associated raw data
- A description of any restrictions on data availability

Data used in this paper are a subset of data collected in the Nutrient Network experiment and are available as source data EDI submission (https://doi.org/10.6073/pasta/f11f2fcf0e56978ce6d96b8b78dcd0d3).

Raw sequence Data deposition: The raw sequence data have been deposited in the NCBI Sequence Read Archive (accession no. SRP052716 and BioProject accession

no. PRJNA272747). The shotgun metagenomic sequences have been deposited in the Genomes Online Database (GOLD Study ID Gs0053063).

In the paper, we include the following statement: Source data (plant, fungal, climate, and soil properties) are available in the Environmental Data Initiative (EDI) repository with the identifier INSERT HERE. Climate data was originally collected from the WorldClim database (version 1.4) is available at http://www.worldclim.org/bioclim.

The WorldClim database (version 1.4) is available at http://www.worldclim.org/bioclim.

# Field-specific reporting

Please select the one below that is the best fit for your research. If you are not sure, read the appropriate sections before making your selection.

☐ Life sciences ☐ Behavioural & social sciences ☒ Ecological, evolutionary & environmental sciences

For a reference copy of the document with all sections, see nature.com/documents/nr-reporting-summary-flat.pdf

# Ecological, evolutionary & environmental sciences study design

All studies must disclose on these points even when the disclosure is negative.

**Study description**

We used a published fungal dataset generated from 25 NutNet grassland sites that are distributed world-wide and where researchers follow the same treatment and sampling protocols. Each grassland site is situated in a relatively homogeneous ~1000 m2 area divided into three blocks. Each block contains 5 x 5 m plots that are surveyed annually for plant community composition and productivity and a sub-set of plots receive applications of 10 g of either N [(NH2)2CO] or P [Ca(H2PO4)2] or both m-2 year-1.

**Research sample**

Data included in this analysis were part of a previously collected data set (Leff et al. 2015) as well as preserved roots from that sample collection. The raw soil fungal sequence data have been deposited in the NCBI Sequence Read Archive (accession no. SRP052716 and BioProject accession no. PRJNA272747). The shotgun metagenomic sequences have been deposited in the Genomes Online Database (GOLD Study ID Gs0053063).

Briefly, soil samples five (2.5cm x 10cm deep) for fungal community analyses were collected and homogenized from individual plots in peak growing season during 2011 or 2012, 1-4 years after the initial nutrient addition, and DNA was extracted and amplified using fungal-specific primers (ITS1F/ITS2) targeting the internal transcribed spacer (ITS1) region. Root biomass and pH were also measured in these cores, and a subset of root biomass was set aside for measurement of fungal colonization.

We matched taxonomic identities of fungal sequences randomly rarefied to 485 sequences per plot with ecological guilds using the expert-curated database FUNGuild28. This rarefaction level represents a trade-off between keeping as many sites as possible in our analyses while characterizing most taxa within each treatment at each site. This rarefaction was also chosen as it was the same as in Leff et al. 2015, thus allowing direct comparisons with that publication. While this rarefaction level did not result in an exhaustive characterization of all taxa at all sites, it did capture abundant taxa in all sites (Fig. S6). We then assessed if sequence numbers belonging to different guilds varied across sites and among control plots and those receiving N, P, or N+P. Of the 164,900 total sequence reads, 60% belonged to taxa annotated with a guild assignment. These assignments were further subset into three guilds: highly probable and probable arbuscular mycorrhizal (AMF, putative mutualists), plant pathogenic fungi, and saprotrophic fungi. For the latter, both the soil saprotroph and undefined saprotroph guilds were included. The specific substrate or habitat of many saprotrophic fungi is undefined, and by only including soil and undefined saprotrophs, we sought to exclude those taxa known to associate with non-representative substrates such as wood or dung. Total sequence counts were 5659 for AMF, 13263 for plant pathogens, and 24333 for saprotrophs.

Roots extracted from the soil cores were used to measure fungal colonization. We collected ~1 g subsample of live roots, clipped them into 2-cm segments, and placed them in Fisher brand Histosette II tis- sue cassettes (Thermo Fisher Scientific, Waltham, MA, USA). We bleached root sub- samples in a 10% KOH solution overnight. We then acidified roots in a 2% HCl solution for 60 min and stained them with 0.05% trypan blue solution overnight (Koske and Gemma 1989). Following staining, we submerged roots in a 1:1 deionized H2O glycerol solution for 3 d to allow any residual trypan blue stain to diffuse from root tissues. We mounted the stained root segments on glass microscope slides using polyvinyl-lacto-glycerol glue (INVAM 2017). We quantified the AMF hyphae by using the magnified intersection method (McGonigle et al. 1990). We inspected a minimum of 50 randomly chosen root intersections on each slide to determined the percentage of AMF colonization.

Plant and soil data used in these analyses were retrieved from the NutNet database on June 3, 2020, but where restricted to years of sampling except for the mediation test where we used average values from control plots spanning 0-4 years since establishment wherever possible. We downloaded climate descriptors from Worldclim v244 to represent long-term historical climate differences among sites, and focused on six non-correlated variables: year average temperature, mean diurnal range, temperature seasonality (standard deviation x 100), total annual precipitation, precipitation seasonality (coefficient of variation), precipitation of warmest quarter and precipitation of coldest quarter. Climatic descriptors were only available at the site level. We also recorded latitude, altitude and elevation of each site to accommodate the spatial correlation used by Worldclim to interpolate the climatic parameters.
Climatic and location predictors were not used for the local models, and edaphic and plant community descriptors were aggregated at the site level for the global models. For the global analysis, we restricted the analysis to control plots to capture the expected global trends in unmanipulated systems and used long-term averages of vegetation predictors to minimize effects of year-to-year variation. In particular, we represented biomass and litter at a site level as across-year averages of the control plots biomass and litter, respectively, soil properties were the mean value of the control plots at the beginning of the experiment, and guild abundances were the mean value of the control plots when the samples were collected.

We estimated biomass and biodiversity measurements of the whole plant community and of each functional group. For the global analysis, we accumulated all the species observed across 5 years in the control plots in each site. Using this aggregated species list we estimated the site-level richness, Faith's phylogenetic diversity (PD), mean phylogenetic distance (MPD) and mean nearest phylogenetic distance (MNTD). We repeated the biomass and diversity measures using species that belong to different functional groups (grasses, legumes and forbs) and obtained biomass and biodiversity estimates by functional group. For the local models, we used biomass and cover data of the year when the fungi soil samples were collected, and estimated the same predictors: richness, PD, MPD, and MNTD, each using the whole community and each functional group. MPD and MNTD were not estimated for legumes, as legumes were absent in some sites and plots. Similar to Leff et al. 2015, we estimated the Jaccard dissimilarity between each plot and the corresponding control plot of the given block in each site. We included litter mass as a predictor for saprotrophs.

Finally, we generated two .Rdata files which consisted of all the fungal FUNGuild data, fungal colonization, plant community parameters, soil properties, and climate data, one for the plot-level analyses, one for the site-level analyses which we submitted with all R scripts to the EDI repository (insert DOI HERE).

Sampling strategy

We included as many as the NutNet sites that were operational at the time and able to collect soil cores using a consistent protocol. Five soil cores (2.5 cm diameter × 10 cm deep) were sampled from two 0.1 × 1 m strips adjacent to each floristic plot, homogenized and shipped on ice to a central processing laboratory (Corvallis, Oregon, USA), then distributed for root biomass, pH, fungal DNA extraction, fungal colonization analyses . To minimize contamination the soil corer was washed with alcohol wipes between plots, and gloves were worn. We chose to collect five 2.5 cm diameter soil cores to minimize disturbance to long-term monitored vegetation plots while allowing us to have a representative plot-level sample to understand broad responses of fungal communities to nutrient addition.

We did not perform any power analysis or similar statistical analyses to determine the proper number of samples that should be targeted because the research infrastructure was pre-established. Additionally, because our analysis relied on pre-collected data and root samples, we were limited in the number of samples that had been collected between the years of 2011 and 2012.

Data collection

Plant community composition and biomass were assessed and soil samples were collected during the growing season of 2011 or 2012 by each of the site-level PIs and research groups. Plant species were identified within the plots, and plant biomass and soil cores were sampled directly adjacent to the plots to determine soil characteristics and assess microbial community structure (8). Soil cores were shipped on ice to a central processing facility (Corvallis, OR) immediately after collection, and samples for microbial community analysis were preserved at −20 °C. Analyses to determine soil pH and C, N, and P content in Elizabeth Borer & Eric Seabloom's lab at the University of Minnesota. Fungal community diversity and composition was assessed by using targeted marker gene surveys focusing on the internal transcribed spacer (ITS) region for Fungi by Jonathan Leff & Noah Fierer. DNA was extracted by inserting a sterile swab into each soil sample and cutting the swab tip off into a well in a bead plate of the PowerSoil-htp 96-well DNA extraction kit (Mo Bio Laboratories, Inc.). Ylva Lekberg and Peter Kennedy matched taxonomic identities of fungal sequences randomly rarefied to 485 sequences per plot with ecological guilds using the expert-curated database FUNGuild database to assess if sequence numbers belonging to different guilds varied across sites and among plots that had received no nutrients, N, P or N+P. Dried roots from the soil cores were cleared in 10% KOH and stained with Trypan Blue during 2018 by Jeremiah Henning and counted using the grid-line intercept method.

Timing and spatial scale

Soil samples for fungal community analyses as well as plant community metrics were collected from individual plots during peak biomass in 2011 or 2012, 1-4 years after the initial nutrient addition. Root and soil collection were conducted as a 1-time sampling at peak biomass when researchers accessed their plots in 2011 or 2012. Because our sites encompass Northern & Southern Hemispheres and a wide range of latitudes, the dates of peak biomass differ site to site. Plant measures are collected annually during peak biomass from within a standardized 1mx1m plots embedded within the larger 5x5m plots, thus plant metrics are consistently conducted year to year.

Data exclusions

We excluded NutNet sites from our analysis that did not collect soil samples initially collected for the Leff et al. 2015 paper. However, we did have an additional 4 sites that root data existed that we did not have sequence data for. Of the 164,900 total sequence reads we obtained, 60% belonged to taxa annotated with a FUNGuild guild assignment. These assignments were further subset into three guilds: highly probable and probable arbuscular mycorrhizal (AMF, putative mutualists), plant pathogenic fungi, and saprotrophic fungi. For the latter, both the soil saprotroph and undefined saprotroph guilds were included. The specific substrate or habitat of many saprotrophic fungi is undefined and by only including soil and undefined saprotrophs, we sought to exclude those taxa known to associate with non-representative substrates such as wood or dung. Exclusion criteria for FUNGuild were pre-established in accordance with author Peter Kennedy who is associated with maintaining the FUNGuild database. Total sequence counts for AMF was 5659, plant pathogens was 13263, and saprotrophs was 24333.

Reproducibility

By conducting the same experimental methodology across 25 unique sites around the globe, we can assess the response of soil fungal guilds to nutrient deposition independent of site-level contexts. Within our statistical models, site is always treated as a random effect and we include as many site-level and plot-level contexts to understand drivers of the patterns we observed. For instance, to parameterize our structural equation model, we compared fungal guild sequences across nutrient addition treatments as a linear mixed-effects model, incorporating site and block within sites as random effects using lmerTest package. We calculated the partial effect of the treatment using the visreg package (Fig 1). Fungal guild sequences were log +1 transformed to meet assumptions of normality. To assess the potential mechanisms driving the treatment effect, we included three sets of covariates to determine if any of them could drive the observed patterns; post-treatment vegetation, post-treatment pH and root biomass, and pre-treatment soil conditions (Table S6). For each guild, we eliminate one of each pair of highly correlated variables identified using variance inflation factor (VIF) and, to obtain a more parsimonious model, we retained only significant variables. Post-treatment vegetation was described using functional group descriptors or whole community descriptors and the best model in each case compared between them. To aid in the reproducibility of our analyses, we included source data along with R-scripts to reproduce SEM, to conduct co-occurrence analysis, and conduct the site-level and global-scale (control plots only) mediation analyses and to generate subsequent tables.

Randomization

When establishing the nutrient addition experiments at a site, nutrient treatments are randomly assigned to plots that are organized into three blocks. When collecting soil cores, we collected samples from consistent spatial locations in each plot to minimize any

collector bias or effects of plant species should be randomly distributed on cores, independent of nutrient addition treatment.

Blinding

Soil cores and the subsequent DNA products as well as the root samples for root colonization data was simply given an independent number system which had no information on either site, block, or nutrient addition treatment. Matching fungal sequence data and root colonization data to the experimental metadata was done on the back end of the experiment.

Did the study involve field work? ☒ Yes ☐ No

## Field work, collection and transport

Field conditions

All 25 sites used in this study were from temperate-zone grasslands in Africa, Australia, Europe, and North America, and all were part of the Nutrient Network experiment and are described. In brief, the sites ranged in many environmental characteristics, including mean annual precipitation (262–1,898 mm·y−1), mean annual temperature (0–18 °C), elevation (50–2,320 m), soil pH (4.5–8.4), total soil P (1–253 ppm), soil %N (0.03–1.5%), and aboveground plant productivity (15–1482 g−2·y−1) (Leff et al. 2015). Plant community composition and biomass were assessed and soil samples were collected during the growing season of 2011 or 2012. Plant species were identified within the plots, and plant biomass and soil cores were sampled directly adjacent to the plots to determine soil characteristics and assess microbial community structure.

Location

| Site name | Continent | Country | Latitude | Longitude | Elev, m |
|---|---|---|---|---|---|
| Bunchgrass (Andrews LTER) | N. America | USA | 44.28 | −121.97 | 1,318 |
| Burrawan | Australasia | Australia | −27.73 | 151.14 | 425 |
| Chichaqua Bottoms | N. America | USA | 41.79 | −93.39 | 275 |
| Cedar Point Biological Station | N. America | USA | 41.2 | −101.63 | 965 |
| Cowichan | N. America | Canada | 48.46 | −123.38 | 50 |
| Elliott Chaparral | N. America | USA | 32.88 | −117.05 | 200 |
| Fruebuel | Europe | Switzerland | 47.11 | 8.54 | 995 |
| Mt Gilboa | Africa | South Africa | −29.28 | 30.29 | 1,748 |
| Hall's Prairie | N. America | USA | 36.87 | −86.7 | 194 |
| Hart Mountain | N. America | USA | 42.72 | −119.5 | 1,508 |
| Konza LTER | N. America | USA | 39.07 | −96.58 | 440 |
| Lancaster | Europe | UK | 53.99 | −2.63 | 180 |
| Lookout (Andrews LTER) | N. America | USA | 44.21 | −122.13 | 1,500 |
| Mt. Caroline | Australasia | Australia | −31.78 | 117.61 | 285 |
| Sagehen Creek UCNRS | N. America | USA | 39.43 | −120.24 | 1,920 |
| Saline Experimental Range | N. America | USA | 39.05 | −99.1 | 440 |
| Shortgrass Steppe LTER | N. America | USA | 40.82 | −104.77 | 1,650 |
| Sheep Experimental Station | N. America | USA | 44.24 | −112.2 | 910 |
| Sierra Foothills REC | N. America | USA | 39.24 | −121.28 | 197 |
| Smith Prairie | N. America | USA | 48.21 | −122.62 | 62 |
| Spindletop | N. America | USA | 38.14 | −84.5 | 271 |
| Summerveld | Africa | South Africa | −29.81 | 30.72 | 679 |
| Ukulinga | Africa | South Africa | −29.67 | 30.4 | 842 |
| Duke Forest | N. America | USA | 36.01 | −79.02 | 141 |
| Val Mustair | Europe | Switzerland | 46.63 | 10.37 | 2,320 |

Access & import/export

Importation of soil samples to the University of Minnesota were conducted under United States Department of Agriculture, Animal and Plant Health Inspection Service permit # PCIP-17-00144, application number P588-170301-002.

Disturbance

Sites are only visited once annually to minimize disturbance.

# Reporting for specific materials, systems and methods

We require information from authors about some types of materials, experimental systems and methods used in many studies. Here, indicate whether each material, system or method listed is relevant to your study. If you are not sure if a list item applies to your research, read the appropriate section before selecting a response.

## Materials & experimental systems

| n/a | Involved in the study |
|---|---|
| ☒ | ☐ Antibodies |
| ☒ | ☐ Eukaryotic cell lines |
| ☒ | ☐ Palaeontology and archaeology |
| ☒ | ☐ Animals and other organisms |
| ☒ | ☐ Human research participants |
| ☒ | ☐ Clinical data |
| ☒ | ☐ Dual use research of concern |

## Methods

| n/a | Involved in the study |
|---|---|
| ☒ | ☐ ChIP-seq |
| ☒ | ☐ Flow cytometry |
| ☒ | ☐ MRI-based neuroimaging |
